# Gold Nanoparticle-Mediated Gene Therapy

**DOI:** 10.3390/cancers14215366

**Published:** 2022-10-31

**Authors:** Gayathri A. Kanu, Javad B. M. Parambath, Raed O. Abu Odeh, Ahmed A. Mohamed

**Affiliations:** 1 Department of Medical Laboratory Sciences, College of Health Sciences, University of Sharjah, Sharjah 27272, United Arab Emirates; 2 Department of Chemistry, College of Sciences, University of Sharjah, Sharjah 27272, United Arab Emirates; 3 Center for Advanced Materials Research, Research Institute of Sciences and Engineering, University of Sharjah, Sharjah 27272, United Arab Emirates

**Keywords:** CRISPR/Cas9 system, zinc finger nucleases, TALENs gene editing, RNA interference (RNAi), antisense oligonucleotides

## Abstract

**Simple Summary:**

Successful gene therapy mainly depends on the fabrication of efficient and nontoxic carriers that can compact genetic materials and adequately deliver them to target cells. Nanoscale gold-mediated gene delivery systems have been considered a powerful tool for gene therapy because of their inherent potential of nontoxicity, high specificity, and therapeutic efficacy. Here, we summarize the updated progress in gene therapy by taking an edge on the unique properties of the gold nanoparticles. Distinct employed by these nano-carriers for gene-silencing and gene-editing are a great deal of optimism for the treatment of human fatal genetic disorders. From other published reviews on gold theranostics, this article discusses the recent advances of gold nanostructures in gene therapeutics for diseases caused by a single gene in humans. The promising advancements employed by these nano-carriers for gene-silencing and gene-editing are a great deal of optimism for the treatment of human fatal genetic disorders.

**Abstract:**

Gold nanoparticles (AuNPs) have gained increasing attention as novel drug-delivery nanostructures for the treatment of cancers, infections, inflammations, and other diseases and disorders. They are versatile in design, synthesis, modification, and functionalization. This has many advantages in terms of gene editing and gene silencing, and their application in genetic illnesses. The development of several techniques such as CRISPR/Cas9, TALEN, and ZFNs has raised hopes for the treatment of genetic abnormalities, although more focused experimentation is still needed. AuNPs, however, have been much more effective in trending research on this subject. In this review, we highlight recently well-developed advancements that are relevant to cutting-edge gene therapies, namely gene editing and gene silencing in diseases caused by a single gene in humans by taking an edge of the unique properties of the AuNPs, which will be an important outlook for future research.

## 1. Introduction

Inorganic functional nanomaterials have emerged as reliable and adaptable nano scaffolds for gene delivery [1,2]. The synthesis of numerous primary proteins in microbes and living cells is the focus of cutting-edge recombinant techniques that have replaced outdated methods and the current trends in bio-nanotechnology. For example, “The Human Genome Project” and its developments in molecular genetics in high-throughput techniques have enabled us to decipher the genetic background of numerous diseases and discover novel therapeutic targets [3]. More focus has been placed on nanomedicine, which has enormous future potential to improve nucleic acid-based treatments, such as gene editing, gene silencing, and viral vectors. These have the potential to significantly advance the treatment of cancer and genetic diseases, such as acute immunodeficiency and Parkinsonism [4].

In principle, gene therapy relies on delivering recombinant synthesized nucleic acids to insert, delete, edit, or silence the gene or gene sequences, as opposed to drugs which produce more susceptible phenotypes. As a result, it is possible to restore cell function in monogenic illnesses or give cells new capacities. However, there are many obstacles to overcome to attain this goal, specifically, the nonspecific capture of gene delivery vehicles by the liver reticuloendothelial system and endosomal trapping, which leads to a marked reduction in the delivery efficiency to the target tissues/cells [5]. Oligonucleotides (e.g., antisense oligonucleotides) and polynucleotides (e.g., messenger RNA) have a relatively limited half-life in physiological fluids due to the presence of endo- and exonucleases [6,7], and this is the major challenge in ensuring they reach their target organs/tissues/cells without degradation.

The construction of safe, biocompatible nano-carriers that can conjugate with genetic materials to transfer therapeutic agents to different cells, tissues, or organs is the most difficult engineering problem encountered while constructing an effective delivery system [8]. Although viral-based nanocarriers are highly effective and widely utilized in immunization, researchers have worked tirelessly to avoid utilizing viruses due to their intrinsic immunogenicity, difficult synthetic pathways, and protein pre-modification [8]. Synthetic methods with minimal or low immunogenicity, high biocompatibility, ease of manufacture, a programmed target ability, and the capacity to be injected numerous times [9,10,11] are judiciously viewed as promising alternatives.

Gold nanoparticles (AuNPs) have been analyzed in detail for decades and are the most developed alternative for a selection of medical applications, such as sensing, imaging, catalysis, therapies, diagnostics, medication, and gene delivery [12,13]. AuNPs have unique features that set them apart from their alternatives [14]. Firstly, AuNPs can be coated with cationic molecules to modulate the surface charge of nanostructures and enhance DNA binding via an electrostatic interaction due to their physicochemical properties. Supporting studies have demonstrated the efficiency of AuNPs as DNA carriers [15,16]. Secondly, the excellent photophysical properties of AuNPs are crucial in bio-diagnostic testing. Thirdly, their simple surface chemistry enables them to function as synthetic antibodies with binding interactions that can be accurately adjusted by varying the density of the binding molecules in their shells. Therefore, gold nanotechnology could make biomedicine a successful route for improving drug delivery by enabling targeted, diagnostic, and therapeutic capabilities that can be chemically customized for a particular condition.

## 2. Gene Editing

### 2.1. CRISPR/Cas9 System 

Genetic disorders previously believed to be incurable may soon be treated, thanks to the “CRISPR-Cas9” system’s ease of use, versatility, precision, and site specificity. The discovery of clustered regularly interspaced short palindromic repeats, the CRISPR-associated nuclease system (CRISPR/Cas9), and the transition of genome engineering from bacterial to mammalian cells have represented a substantial change. This method of genome engineering has been used not only in different cell lines but also in human primary and stem cells. Moreover, in vivo experiments have used zebrafish and mice to investigate gene functions, cancer models, and gene therapy techniques [17,18,19,20,21,22,23]. Thus, the CRISPR/Cas9 method has the potential to alter how genetic diseases are treated [24,25]. Recently, treatment of the most common genetic diseases, such as cancer, have been focused on CRISPR/Cas9-based genetic approaches. However, there are a few more on the list, such as Duchenne’s muscular dystrophy, cystic fibrosis, Leber congenital amaurosis, thalassemia, sickle cell disease, and Huntington’s disease, which could be cured or treated using similar genetic approaches [26,27].

**Figure 1:**
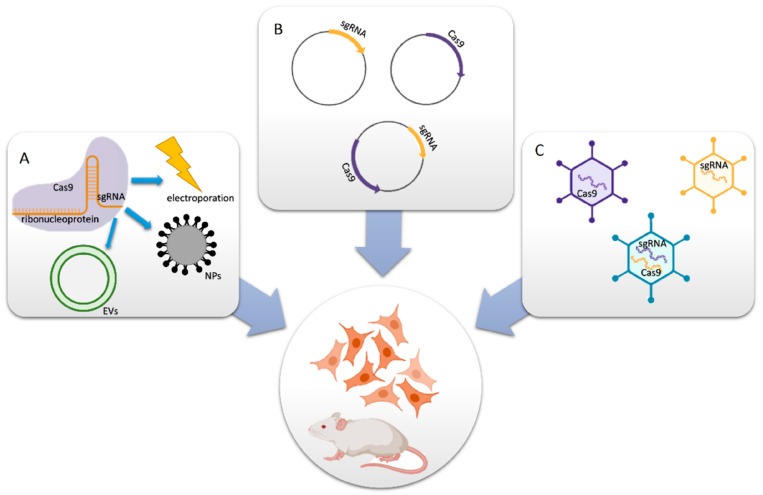
Delivery methods for CRISPR-Cas9 components. (**A**) Cas9 protein and sgRNA form an RNP complex for packaging purposes. (**B**) Cas9- and/or sgRNA-expressing plasmids are transferred into cells. (**C**) In vitro or in vivo viral vectors encoding Cas9 and/or sgRNA convey the mechanisms (reprinted with permission from [34]).

There are two main components of a well-designed CRISPR-Cas9 system; the first is a single-guide RNA (sgRNA), and the second is Cas9 endonuclease. If necessary, the system can also include a donor template for homologous repair. Together, these two elements create active ribonucleoprotein (RNP) complexes that may pinpoint a precise genomic region where double-strand breaks (DSBs) are created because of editing [28,29,30]. The application targeted delivery system specific to the disease, especially the CRISPR-Cas9 technique, requires an efficient carrier method or molecule for sufficient cellular uptake and protection against the degradation of the protein that finally leads to the on-/off-target effects of a particular disease [31]. Chemical methods or synthetic transfection carriers have the advantage that their properties may be tailored to meet the needs of delivering CRISPR systems in gene correction. Additionally, they help overcome the limitations of biosafety, loading, and packaging capacities. Non-viral vectors, in comparison with viral vectors, possess low immunogenicity, lack endogenous virus recombination, reduce restrictions in delivering larger genetic payloads, and are simpler to manufacture on a large scale. Non-viral vectors may offer enticing prospects for CRISPR-Cas9 delivery. This is the reason why non-viral vectors are considered an alternative for CRISPR-Cas9 gene therapy translational studies [32,33,34]. A schematic representation displaying the CRISPR-Cas9-based delivery system is shown in Figure 1.

**Figure 2:**
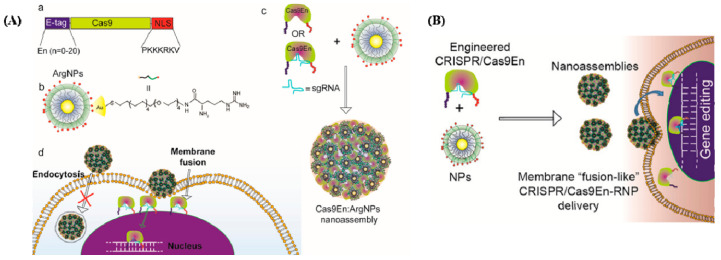
(**A**) Cas9/sgRNA delivery using AuNPs and gene editing. (**a**) Cas9 protein engineered with ArgNPs for intracellular delivery of Cas9-protein/RNP through membrane fusion. Illustration of Cas9 transporting an E-tag at the N-terminus and a nuclear localization signal at the C-terminus (NLS). (**b**) Chemical structure of ArgNPs. (**c**) Nano assembly of Cas9En-RNP and ArgNPs. (**d**) A membrane fusion method for delivering Cas9En (reprinted with permission from [35]). (**B**) Conjugation of Cas9 nuclease and carrier NPs makes CRISPR component packing and transport more efficient. Cas9En self-assembled into massive nano assemblies with arginine-functionalized-AuNPs (reprinted with permission from [35]).

The shortest co-delivery method for Cas9 protein and sgRNA was developed by Mout et al., representing the first use of materials based on AuNPs in genome editing (Figure 2A [35]). The proposed Cas9 protein was used to interact with cationic arginine-AuNPs, sgRNA, and a negatively charged glutamate peptide tag. AuNPs and delivery payloads interacted to create nano assemblies that could quickly bind to cell membranes and release compressed Cas9 protein and sgRNA cargo into the cytoplasm. Up to 90% of target genes were successfully transfected using this method, which also had good gene editing efficiencies, such as adeno-associated virus integration site 1 (AAVS1) (29%) and phosphatase and tensin homolog (PTEN) (up to 30%) in in vitro investigations.

Lee et al. developed a CRISPR–AuNPs vehicle for the direct transfer and quick discharge of Cas9 ribonucleoprotein and donor DNA repair templates for correcting gene alterations in model for studying Duchenne muscular dystrophy (MDX) mice [36]. AuNPs of 15 nm were first conjugated with thiol-modified oligonucleotides, and then crossed with donor DNA and loaded with a Cas9–RNP complex, and finally decked with a cationic polymer, poly{*N*-[*N*′-(2-aminoethyl)-2-aminoethyl]aspartamide}, PAsp (DET). PAsp (DET) on the exterior of CRISPR–AuNPs can cause endosomal distraction and enable endosomal escape at the end of cellular endocytosis, releasing it into the cytoplasm and causing a release of the Cas9–RNP complex and donor DNA from the CRISPR–AuNPs. An exterior coating of PAsp (DET) facilitates the endosomal escape. Increased intracellular levels (~10 mM) of glutathione enable the release of the Cas9–RNP complex and donor DNA from CRISPR–AuNPs. This made it possible to restore the functional wild-type sequence in MDX dystrophin-deficient mice, which reduced muscle fibrosis, restored dystrophin protein expression, and restored muscular function without completely altering inflammatory cytokines. CRISPR–AuNPs, used together, present a possible healing method for the active treatment of DMD patients by eliminating mutant genes.

A typical single-gene form of autism spectrum disorder was used in a mouse model of Fragile X Syndrome. Lee et al. used CRISPR–AuNPs as the carrier to transfer RNP to the brain and helped the mouse recover its high repetition of behaviors [37]. Following the intracranial injection of CRISPR–AuNPs targeting the mGluR5 gene in the striatum of wild-type or Fmr1 knockout mice, the local mGluR5 gene levels in the striatum were successfully reduced.

AuNPs have been studied in conjunction with their photothermal properties and the traditional benefits of lipid-based particles [38], in which cationic AuNPs were formed by joining short peptides. This compound accelerates cellular uptake by TAT-peptides of AuNPs. The polo-like kinase 1 (PLK-1)-targeting Cas9–sgRNA plasmid was then coupled with the cationic AuNPs. The cationic lipid mixture of 1,2-dioleoyl-3-trimethylammoniumpropane, DOPE dioleoyl phosphatidylethanolamine, and cholesterol was next placed onto the AuNP–plasmid, and the particles were further enhanced by adding 1,2-distearoyl-sn-glycero-3-phosphoethanolamine-poly(ethylene glycol) and 2-distearoyl-sn-glycero-3-phosphoethanolamine-poly(ethylene glycol) to boost permanency. It was confirmed that PLK-1 disruption in melanoma cells was enhanced by light-irradiated AuNPs.

The flexible cationic AuNP platform known as arginine ArgNPs was developed for improved membrane transfer [35,39]. Engineers used Cas9 with a glutamate peptide tag and NLS because glutamic acid generates a negative potential around the Cas9 RNP to connect to the positive ArgNPs. In contrast to endocytosis, it was discovered that Cas9–ArgNPs were internalized by cells via a membrane-fusion-like procedure that required cholesterol. Furthermore, cytosol delivery was related to the chain length of the glutamate peptide used. In HeLa cells, Cas9–ArgNPs targeting the PTEN or AAVS1 genes caused a 30% indel, demonstrating gene editing. Another study showed the flexibility of this delivery method by using Cas9–ArgNPs to produce SIRP knockout RAW264. 7 macrophages to enhance the phagocytosis of cancer cells [40].

SpCas9–AuNCs were localized into cells through endocytosis and subjected to endosomal and lysosomal low pH, which caused the disassociation of the SpCas9–AuNCs as well as the proton sponge, resulting subsequently in the release of the SpCas9 into the cytoplasm [41]. The SpCas9–AuNCs were then used to see if they could edit the E6 gene in the HPV 18 genome in HeLa cells using an E6 sgRNA transfection, and the expression of HPV18 E6 protein was dramatically decreased after treatment with SpCas9–AuNCs.

Mout et al. designed the Cas9 protein to have a negative charge, resulting in a protein that resembled sgRNA electrostatically [35]. In this research, an oligo glutamic acid tag (E-tag) was fused to the N-terminus of a Cas9 protein, Cas9En (n: glutamic acids), and the sgRNAs were assembled with cationic arginine AuNPs (ArgNPs) and delivered directly to the cytoplasm and nucleus (Figure 2B).

### 2.2. Zinc Finger Nucleases (ZFNs) and TALENs Gene Editing

ZFNs were first discovered in Xenopus oocytes as a component of transcription factor IIIa [42], and they were subsequently identified as a site-specific endonuclease for cutting DNA [43]. ZFNs have site-specific DNA binding properties. They possess a group of Cys2His2 zinc fingers (ZFs) created via the interaction between their ZF-domains and similar DNA regions. ZF proteins are a type of transcription factor that is connected to an endonuclease. Each ZF unit specifically identifies three DNA base pairs and generates base-specific connections interacting with the major groove of the DNA [44,45] through its helix residues.

**Figure 3:**
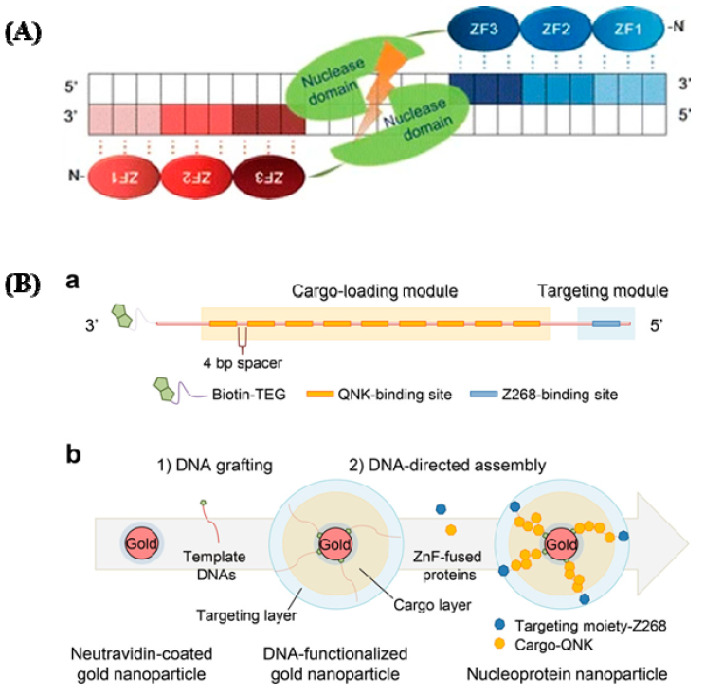
(**A**) ZFNs have a specific structure. ZFNs linked to their target locations are depicted schematically. This is a well-designed ZF DNA domain fused to the FokI type IIS restriction enzyme’s DNA cleavage domain that forms nucleases. Each ZFN protein has three ZFs (shaded ovals) that recognize a target sequence in the DNA (shaded boxes). The binding of two ZFNs on target DNA with a spacer (5/6 bp) in the center causes the ZFN nuclease domains to dimerize and the target DNA to be cleaved (reprinted with permission from [51]). (**B**) DNA and zinc fingers are used to program the construction of nucleoprotein nanoparticles. (**a**) Template modular DNA design. A biotin molecule was added at the 3′ end of the template DNA with a TEG linker for grafting template DNA onto AuNPs. To eliminate steric hindrance, multiple binding sites for QNK-QNK-RHR (QNK) were spatially organized with a four-base-pair spacer. (**b**) Sequential construction of nucleoprotein nanoparticles is depicted schematically (NNPs) (reprinted with permission from [52]).

They can bind with any triplet in its naturally occurring setting, which varies depending on tissue condition, protein context, and DNA sequence. Single zinc fingers, which have amino acids [46,47], are straightforward structures with an unusually high degree of functional flexibility and structural malleability. The effectiveness of HR has been greatly boosted by the introduction of ZFNs to create gene-specific DNA breaks [48]. A FokI type II RE creates the DNA cleaving domain, then dimerized to specifically target its corresponding regions [49]. Two exogenous protein domains are active: the first one is a restriction nuclease called FokI, and the second one is a sequence-specific zinc-finger transcription factor protein (ZFP). FokI, a restriction nuclease enzyme, detects trinucleotide DNA sequences, sequence specific ZFPs, and FokI. The nuclease domain, which is composed of a wide array of C2H2 zinc fingers, interacts with this domain, limiting the DNA-binding specificity of the ZFNs and directing them to their target site (Figure 3A). The ZFN gene-repair process is as follows: identification of the full ZFN-binding site within the target GOI (gene of interest); design of a pair of ZFNs; testing of the ZFN pair for activity; identification of a targeting construct to produce the anticipated genomic modifications; and co-transfer of the ZFNs and the targeting vector to produce the cuts and insertion of the therapeutic transgene to the GOI [50]. In Figure 3B-a, cellular targeting moiety and a cytotoxic protein payload, respectively, were genetically coupled to two forms of ZnF that recognize various DNA sequences. Using streptavidin-biotin chemistry, double-stranded DNA with many ZnF-binding sites was created and grafted onto the gold nanoparticles. To create the assembly of nucleoprotein nanoparticles, the ZnF-fused proteins and DNA-functionalized nanoparticles were co-incubated.

ZFPs are found in significant quantities in the genomes of various organisms; in humans, they account for 3% of the overall genome and are very prevalent DNA-binding motifs in transcription factors. With the significant recent advances [51,52], the roles of ZFPs in the health sector are gradually being unraveled. They are becoming recognized targets for treating cancers [53,54], viral infections [55], and, more recently, neurological disorders. Fairall et al. observed the significance of metal compounds displacing Zn (II) from ZFPs [45]. With a better understanding of how metal complexes interact with ZFs, innovative metallic compounds have been created to target specific ZFs via coordination and organometallic chemistry. For precise modulation of intracellular signaling pathways, a variety of protein delivery techniques have gained attention [56,57,58,59,60,61,62,63,64,65,66]. Metallic NPs are the initial special materials for creating a nanocarrier due to their distinctive nanoscale dimensions and morphologies. Nucleoprotein-layered AuNPs using DNA and ZnFs were investigated for targeted protein delivery in xenograft mouse models [67], and this showed their utility and potential by demonstrating competent and tumor-selective cytosolic transport of the protein cargo, as well as remarkable tumor regression with negligible toxicity (Figure 3B). Another group studied nanoparticle clusters as an improved MRI contrast agent and dual probe for magnetic and fluorescence imaging using HeLa cells to examine the overexpression of folate receptors [68]. 

**Figure 4:**
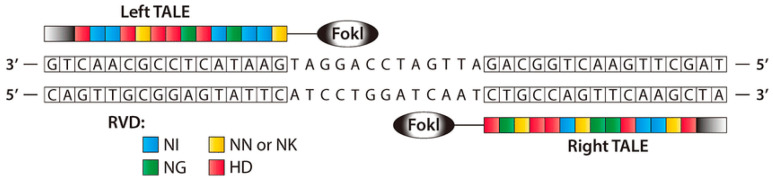
A TALEN dimer coupled with target DNA is shown schematically. To develop a modified nuclease that can detect unique left and right half-sites, TALE arrays are fused to the FokI nuclease domain. A spacer 12–20 bp in length separates the two TALE binding sites (reprinted with permission from [74]).

Like ZFNs, TALENs are naturally occurring protein nucleases that cause DNA DSBs by fusing a non-specific DNA cleavage domain with a sequence-specific DNA-binding domain. A highly conserved repeat sequence from transcription activator-like effector (TALE), a protein originally discovered in phytopathogenic Xanthomonas bacteria, that spontaneously alters gene transcription in host plant cells, makes up this “DNA-binding region” [69,70]. ZFNs and TALENs are structurally and functionally identical, with TALENs being more specific, although both contain the restriction endonuclease Fok [69]. To bind to a specific DNA sequence, TAL effector proteins must be engineered. TALE binds to DNA through a core section containing a collection of three-amino-acid (33–35) motifs (Figure 4). A typical TALE DNA-binding domain recognizes 1420 nucleotides, including the conserved thymine (T) base at the 5′ borders. The repeat-variable di-residues (RVDs) [70,71] are two hypervariable amino acids that determine TALE selectivity. The RVDs are conserved at positions 12 and 13 [72]. There are around 24 known distinct RVDs, and these RVD loops contain an amino acid repeat with a missing residue denoted with N* means, 33 [73] being the most prevalent. It is easier to assemble four basic components that can recognize the bases A, T, G, and C in TALENs than it is to assemble ZFN subunits that detect 64 different DNA trio or triplet pairings. ZFNs and TALENs, on the other hand, rely on highly precise protein–DNA interactions that allow for fewer mismatches [74].

By inserting an NHEJ/HDR-induced change in a coding region, TALENs were able to disrupt the genes NTF3 and CCR5 in human leukemia cells, indicating that TALENs are designed for endogenous gene cleavage [75]. Breaking a single allele from the Fms-related tyrosine kinase 3 (FLT3) gene using a site-specific TALEN lead to the creation of isogenic leukemia cell clones. The use of artificial TALENs in prostate cancer cells and androgen receptor (AR) target gene reorganizations are functionally categorized sources of resistance [75]. Besides studies revealed that the type of gene-editing technology is a potent and broadly appropriate platform for examining gene transmutations on a molecular basis. This technology has been used in cancer cells such as prostate cancer cells, breast cancer cells [76], and hepatocellular carcinoma cells (HCC) [77] to knock out the genes. However, due to the numerous limitations of ZFN and TALEN, there has been limited or no research on nanomedicine applications.

## 3. Gene Silencing

### 3.1. RNA Interference (RNAi)

A groundbreaking study in the nematode *Caenorhabditis elegans* revealed that RNAi is an evolutionarily conserved gene-silencing mechanism [78,79]. RNA interference (RNAi) is cellular machinery that targets messenger RNA and suppresses translation to silence sequence-specific genes [80]. In this mechanism, the antisense region of ds-RNA directs the RNA-induced silencing complex’s recognition and catalytic degradation of a target mRNA. The antisense strand of a double-stranded RNA duplex mediates one type of siRNA (short interfering RNA) which directs the RNA-induced silencing complex’s (RISC) identification and catalytic degradation of the mRNA target. Double-stranded RNA sequences called siRNAs are integrated into the RISC [81,82,83,84,85]. A guide (antisense) strand and a passenger (sense) strand make up a 21-nucleotide siRNA duplex. Argonaute2 (Ago2) is an endonuclease that removes the sense/passenger strand. Another is carried out by endogenous 20–25 nt short RNAs called microRNAs (miRNAs), which either promote or suppress the transformation of their mRNA targets [85]. A promising method of sequence-specific gene silencing is RNAi gene therapy, which can treat serious human illnesses such as genetic disorders, viral infections, and cancer [86,87,88,89]. There is a lot of interest in this technology and in deepening our grasp of both routes’ basic principles to utilize them for medicinal purposes because bringing small RNAs into cells to regulate gene expression has been difficult [90]. The passage of these RNAs through the cell membrane is challenging due to their negative charge and molecular weight of approximately 13 kDa. Further, in the presence of RNAse in the serum, siRNA is more sensitive to breakdown, necessitating the use of a carrier molecule to achieve the aim. siRNA may be complexed or conjugated with a variety of transporters, and our understanding of how siRNA is delivered has advanced [91].

Due to their therapeutic potential, applications of AuNPs in numerous facets of nanomedicine have been investigated, and they are one of the most advanced platforms in gene delivery [92,93]. AuNPs can be loaded and combined with negatively charged gene drugs [94], provided that the core is coated in a layer-by-layer shell of biodegradable polymers. With the aid of various surfactants, AuNPs are often produced in aqueous solutions, where they are then adsorbed or deposited onto the surface [95].

In a study of cationic phospholipid-coated gold nanorods as distribution systems for nucleic acid payloads, when negatively charged DNA, RNA, or siRNA oligonucleotides were attached to a positively charged phospholipid surface, internalization of the AuNRs was observed using darkfield scattering microscopy [96]. Conde et al., starting with cultured human cells and later using invertebrate and vertebrate (mouse) models, used AuNP compositions for effective RNAi delivery. This resulted in the silence of the c-myc protooncogene [97]. The main idea behind this combination is the covalent attachment of nucleic acids to AuNPs, which did not impair their biological activity [98]. Graczyk et al. showed that the CopGFP gene, which was expressed in a model cell line and was the target of a new trimer (AuNPs associated with structural RNA nanoparticles (tectoRNAs) and carrying regulatory siRNA fragments), could be regulated effectively by the new generation trimer as well as a trimer–AuNPs complex, targeting three chosen areas of mRNA. Their tectoRNA–AuNPs constructs have low cytotoxicity in cancer and non-cancerous cell types, indicating that they are biocompatible and may be discharged in the cytoplasm, as seen in transmission electron microscopy images [99].

By showing that anti-eGFP siRNA, pEGFP-N1, or pDsRed-Max-N1 could be stacked for co-delivery on AuNPs using the layer-by-layer self-assembly procedure, Bishop et al. studied the ability of AuNPs to deliver nucleic acids [96,100,101]. Multivalent deoxyribozyme “10–23” AuNPs (DzNP) conjugates were fabricated with varying DNA density, linker length, enzyme orientation, and linker composition to examine the impact of the catalytic effects of the steric environment and gold surface chemistry. They demonstrated that DzNPs can inhibit the expression of GDF15 in cells via a catalytic mechanism separate from RNA interference. A dual DzNP and siRNA–AuNP technique has potential because DNAzyme action is complementary to siRNA-based gene regulation, particularly for those intended to focus on genes that influence RISC function [100]. On the other hand, gene silencing was examined with multilayered siRNA–AuNPs by measuring luciferase activity in MDA-MB231-luc2 cells (sRAuNPs). This could be related to the delayed breakdown of the polypeptide, poly-l-lysine (PLL), which means that the integrated siRNA release has a longer gene-silencing effect [101].

**Figure 5:**
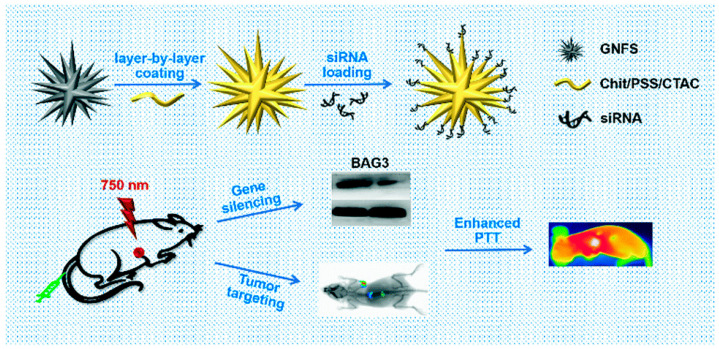
A schematic representation showing the preparation of “GNFs–siRNA” and the treatment process (reprinted with permission from [102]).

By lowering the quantities of associated proteins and mRNA, Liu et al. investigated whether using gold nanoflowers (GNFs) and siRNA (GNF-siRNA pool) can achieve in vitro and in vivo the therapeutic benefits of photothermal treatment and gene silencing. These sequences were used in the silencing of BAG3 genes and lowered systemic toxicity. Furthermore, they experimentally proved that “GNFs–siRNA” could facilitate cellular internalization and induce the endosomal/lysosomal escape of siRNA, resulting in protein and mRNA downregulation and signifying that it would be a suitable vector for siRNA transfer for actual PTT management [102] (Figure 5).

Shim et al. synthesized an aggregate of siRNA coupled to numerous AuNPs using an acid-sensitive ketal linker group. AuNPs and oligonucleotides were released when the ketal linker broke at low pH. Targeted gene silencing was demonstrated by in vitro microscopy studies on GFP-expressing cells under low pH conditions [103]. 

**Figure 6:**
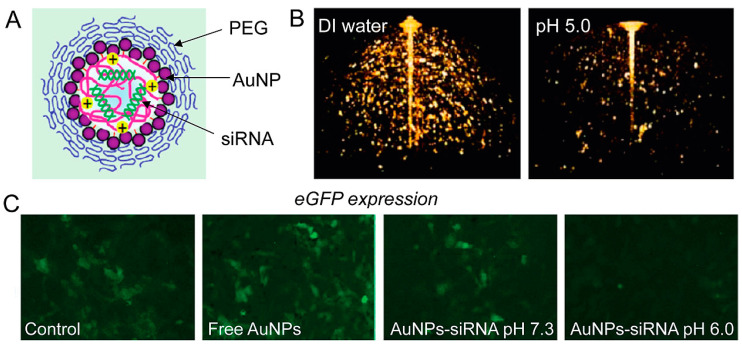
(**A**) AuNPs in imaging and gene-silencing NP design. The siRNA payload is released when the nanoparticle is hydrolyzed at a low pH. (**B**) Three-dimensional optical coherence tomography pictures of AuNP-based nanoparticles (the lower intensity image on the right confirms NPs’ disintegration at lower pH). (**C**) When eGFP-expressing NIH 3T3 cells are treated with AuNPs–siRNA at low pH, fluorescence microscopy demonstrates gene silencing (reprinted with permission from [103]).

Chitosan (CS) and siRNA-coated AuNPs (siRNA/CS–AuNPs) were synthesized using a unique layer-by-layer self-assembly approach. siRNA was shielded from enzymatic degradation by the layer-by-layer self-assembly production of siRNA/CS–AuNPs, maintaining siRNA activity [102]. To integrate optical imaging with gene therapy, Shim et al. conjugated tiny AuNPs to the surface of siRNA-carrying polyplexes using acid-degradable connections. This unique agnostic drug adjusts its photophysical qualities when a cancer-specific stimulus is present. Simultaneous electromagnetic signal alterations and gene silencing in vitro with specific tumor pH-mimicking conditions are used to achieve integrated cancer diagnosis and therapy (theranostics). This innovative type of stimuli-responsive nano theranostics represents an innovation for cancer imaging and treatment that is targeted, multimodal, and combined [103] (Figure 6).

### 3.2. Antisense Oligonucleotides (ASOs)

It was first discovered in a work by Stephenson and Zamecnik where 13-mer generated ssASOs target Rous sarcoma virus mRNA [104,105,106,107], resulting in a translational block [107]. According to several studies, active ASOs are typically 15–20 nucleotides long and have exceptional specificity and affinity to almost any complementary RNA sequence, whether pre-mRNA, mRNA, ribonuclear proteins, or miRNAs, and that they downregulate targeted genes [105] by basic base pairing without significantly increasing far-off toxicity [106]. Further investigation has shown that the two processes that lead to the synthesis of ASOs work as follows: either (1) RNA damage or (2) RNA obstruction. 

**Figure 7:**
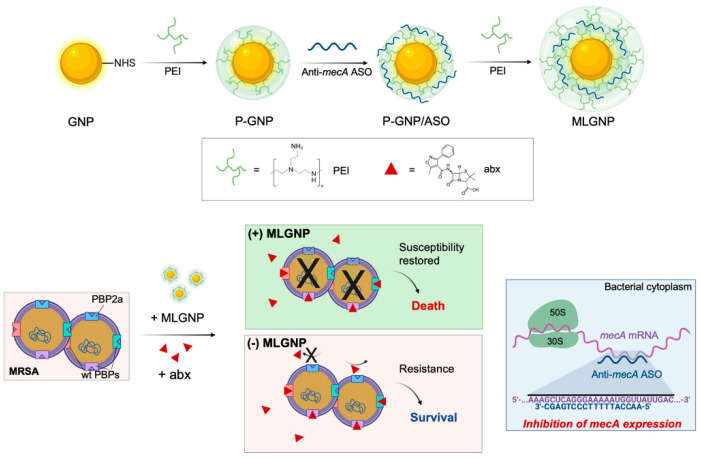
A schematic depiction of how to make ML-AuNPs that deliver antibiotic resistance-targeting ASOs and how to use them to treat MRSA infections in combination (reprinted with permission from [120]).

Antisense treatments are expected to be successful in treating a range of illnesses, including cancer and HIV/AIDS [107]. The FDA has approved ASOs for the treatment of eleven genetic diseases, and many more are in development. AONs have been approved by many countries’ health departments such as the European Medicines Agency (EMA), and the FDA in the United States, with most obtaining commercial approval [107,108,109]. ASOs have had difficulty developing into effective therapeutic systems due to problems related to steady transfection, access to different cell kinds, toxicity, and efficacy [110]. Various non-viral agents, such as cationic lipids and polymers, modified viruses, dendrimers, liposomes, and nanoparticles [111,112,113] have been created to deliver nucleic acids to cells, but each approach has its own set of restrictions. AONs must also have favorable safety, pharmacokinetic, and pharmacodynamic profiles, as well as nuclease resistance [114]. The antisense oligonucleotide is protected from RNase destruction by AuNPs, which increases the half-life and, as a result, the cargo of healing agents supplied to cells. For a variety of nucleic acid moieties, including antisense single-stranded DNA, the ability of AuNPs to vectorize actuators for gene silencing by simple assembly onto the nanoparticle core has been established in vitro and in vivo [114,115,116,117,118]. PBP2A is a selective antimicrobial which restores the susceptibility, causing virulence by the expression of the penicillin-binding protein 2a (PBP2a), considered a transpeptidase encoded by the mecA gene [119]. As shown in Figure 7, Beha et al. introduced multi-layer coated AuNPs (ML-AuNPs) for delivering ASOs targeting the resistance gene of methicillin-resistant *Staphylococcus aureus* [120].

A single-stranded DNA oligonucleotide designed by Vinhas et al. [121] selectively targeted the K562 cells’ expression of the e14a2 BCR-ABL1 gene. The genes were very well silenced by the AuNPs, which significantly increased cell mortality. Changes in the expression of the proteins BCL-2 and BAX, a rise in caspase-3 activity, and the presence of apoptotic bodies in cells treated with the nano conjugate are indicative of the capacity of the compound to cause apoptosis in K562 BCR-ABL1-expressing cells. Furthermore, the imatinib IC50 [122] was decreased when silencing gold nanoconjugate was combined with it.

Kheirandish et al. [122] blocked the *Leishmania major* PTR1 gene using an antisense plasmid. The PTR1 gene was silenced by antisense mRNA. A novel class of non-coding RNA that is solely expressed in the intracellular amastigote stage was discovered by Dumas et al. [123]. Phosphorothioate oligonucleotides were used by Ramazeilles et al. [124] to specifically target the mini-exon sequence, which is present at the 5′ ends of every parasite mRNA and has previously shown that bare AuNPs have antileishmanial properties. The *L. major GP63* gene was silenced. The absorption of ASOs by *L. major* has been observed. According to the study, hybridized AuNPs may kill promastigotes and render the GP63 gene inactive. Jebali et al. [125] concluded that antisense oligonucleotide sequence and size were important for the transfection of *L. major*, which causes cutaneous leishmaniasis. It is important to note that these ASOs can be made from both the coding and non-coding regions of the *GP63* gene. In addition, hybridized AuNPs could kill *L. major* in addition to silencing the *GP63* gene. A detailed review was published on protein degradation pathways in hematological malignancies, in which the specific number of stress categories which lead to protein degradation and unsuccessful treatment was studied [126]. Additionally, AuNPs could potentially aid in the study of genetic diseases. As was already indicated, substantial research on the absorption of important signaling pathways using MG-63 cells with gold–aryl NPs has been conducted by Abdulwahab et al. [127]. They suggested that these particles may be suitable for drug delivery and tracing studies during development. Because they did not affect the biological processes of osteosarcoma cancer cells, these gold–aryl NPs may be effective for drug administration in patients with the disease. This is a key lead for further research in hereditary diseases.

## 4. Future Perspective 

Although both preclinical work and clinical trials focusing on curative therapies are proceeding globally, the clinical translation of CRISPR/Cas9-mediated gene correction or other gene correction is associated with unpredictable outcomes [128]. However, they show promise for medical research, and, importantly, several clinical trials are currently in phase I or phase II. It is noteworthy that therapeutic gene-editing technology is now extending to the ethically contentious matter of changing the genome of human early-stage embryos to protect them from HIV infection [129]. This gives researchers a great deal of optimism for the treatment of human fatal diseases.

## 5. Conclusions

AuNP-mediated gene therapy has already shown great potential in generating disease models for correcting and silencing monogenic disease mutations. The study of the special and unique nature of AuNPs will make it much easier to use them for delivering cargo or correcting gene mutations. However, detailed study and optimization are needed to produce a precise outcome. The novel methods shown here are evidence of the many promising advantages of employing nano-carriers for gene-silencing and gene-editing applications. As suggested by previous research, we have demonstrated how active targeting techniques based on multifunctional nanoparticles, in terms of co-delivery of other medications, trapping of the AuNPs in organic composites for protection or targeted delivery, and photothermal-triggered release, for instance, can be applied in gene therapy. However, we still need more in-depth research and to analyze its results before we can attain tangible outcomes.

## Data Availability

No new data were created or analyzed in this study. Data sharing is not applicable to this article.
